# Polycomb repressive complex 2 regulates sexual development in *Neurospora crassa*

**DOI:** 10.1128/mbio.01505-25

**Published:** 2025-09-30

**Authors:** Abigail M. Deaven, Abigail J. Ameri-Solanky, Zachary A. Lewis

**Affiliations:** 1 Department of Microbiology, The University of Georgia 1355, Athens, Georgia, USA; Universite de Neuchatel, Neuchatel, Switzerland

**Keywords:** *Neurospora crassa*, perithecium, sexual development, Polycomb repression, chromatin modification, H3K27me3

## Abstract

In most branches of the fungal kingdom, sexual development marks a dramatic shift from simple multicellular hyphae to complex fruiting bodies composed of multiple tissues and cell types. It is essential to tightly regulate development to prevent unnecessary energy investment into building these structures. Polycomb repressive complex 2 (PRC2) is a highly conserved regulator of development in multicellular organisms. In plants, animals, and some fungi, PRC2 tri-methylates histone H3 lysine 27 in promoters and gene bodies to repress gene expression. In *Neurospora crassa*, H3K27me3-associated genes are poorly conserved and stably repressed in standard lab conditions. Through analysis of publicly available RNA-seq experiments, we found that PRC2-methylated genes are specifically activated during sexual development. PRC2-methylated genes comprise a distinct subset of developmentally induced genes (DIGs) characterized by an exceptionally high degree of cell-type specificity. Loss of PRC2 activity results in the precocious formation of perithecia-like structures (dubbed false perithecia), even in the absence of a compatible mating-type partner. These structures show a unique gene expression profile with the activation of both PRC2-methylated and unmethylated DIGs, suggesting that loss of PRC2 leads to a major transcriptional reprogramming event. Together, these data suggest that PRC2 is part of a developmental checkpoint that restricts fruiting body development to the appropriate conditions.

**IMPORTANCE:**

Development of multicellular eukaryotes involves transcriptional reprogramming to drive cell fate transitions. This study identified PRC2 as a critical regulator of cell fate in the model filamentous fungus *Neurospora crassa*, where it silences a subset of sexual development genes. Loss of regulation by PRC2 triggers a major reprogramming event in which genes specifying sexual tissues cannot be repressed, causing a homeotic transition. These results provide novel insights into the role of PRC2-mediated regulation in the fungal kingdom and uncover a critical checkpoint regulating complex multicellular development.

## OBSERVATION

Across eukaryotes, key developmental transitions are regulated by transcriptomic, proteomic, and epigenetic regulatory pathways. A central component in this regulation is Polycomb repressive complex 2 (PRC2)**,** a conserved chromatin modifier that catalyzes tri-methylation of histone H3 lysine 27 (H3K27me3) to stably repress genes (1). In plants, insects, and animals, defects in H3K27me3-dependent repression are linked to homeotic transformations, underscoring PRC2’s essential role in organismal development (2–5). Despite its well-established role in the development of higher eukaryotes, the biological significance of PRC2-mediated repression is poorly understood in fungi.

PRC2 and H3K27me3 are absent from the widely used model yeasts, *Saccharomyces cerevisiae* and *Schizosaccharomyces pombe,* but genes encoding PRC2 components are conserved in many fungal lineages (6). The first functional studies of a fungal PRC2 complex were carried out in *Neurospora crassa,* where approximately 7% of genes are enriched for PRC2-dependent H3K27me3 and are tightly repressed in mycelia grown in standard conditions (7). Genetic studies have shown that repression depends on the histone variant H2A.Z (8), the chromatin remodeler Imitation Switch (ISW) (9, 10), the H3K27me3 reader EPR-1 (11), the RPD3L complex (12), the PRC2 accessory subunit (PAS) (13), and components of the constitutive heterochromatin pathway (14–16). While mechanistic insights into H3K27me3-dependent repression have been gained, the biological functions of PRC2 and PRC2-repressed genes in *N. crassa* have remained poorly understood for more than a decade. H3K27me3-enriched domains are overrepresented for genes that are species-, genus-, or ascomycete-specific and most lack conserved functional domains (7). In addition, PRC2-deficient mutants exhibit normal vegetative growth rates, asexual spores (conidia), sexual fruiting bodies (perithecia), and ascospores (7). While the functions of H3K27me3-enriched genes in *N. crassa* are unknown, work in other fungal systems such as *Verticillium dahliae, Epichloë festucae, Magnaporthe oryzae*, and *Fusarium* species has linked H3K27me3 and PRC2 components to diverse processes including plant pathogenesis, symbiosis, and secondary metabolism (17–24). To understand the diversity of PRC2-dependent gene regulation within the fungal kingdom, we sought to investigate functions of PRC2-repressed genes in *N. crassa*.

**Fig 1:**
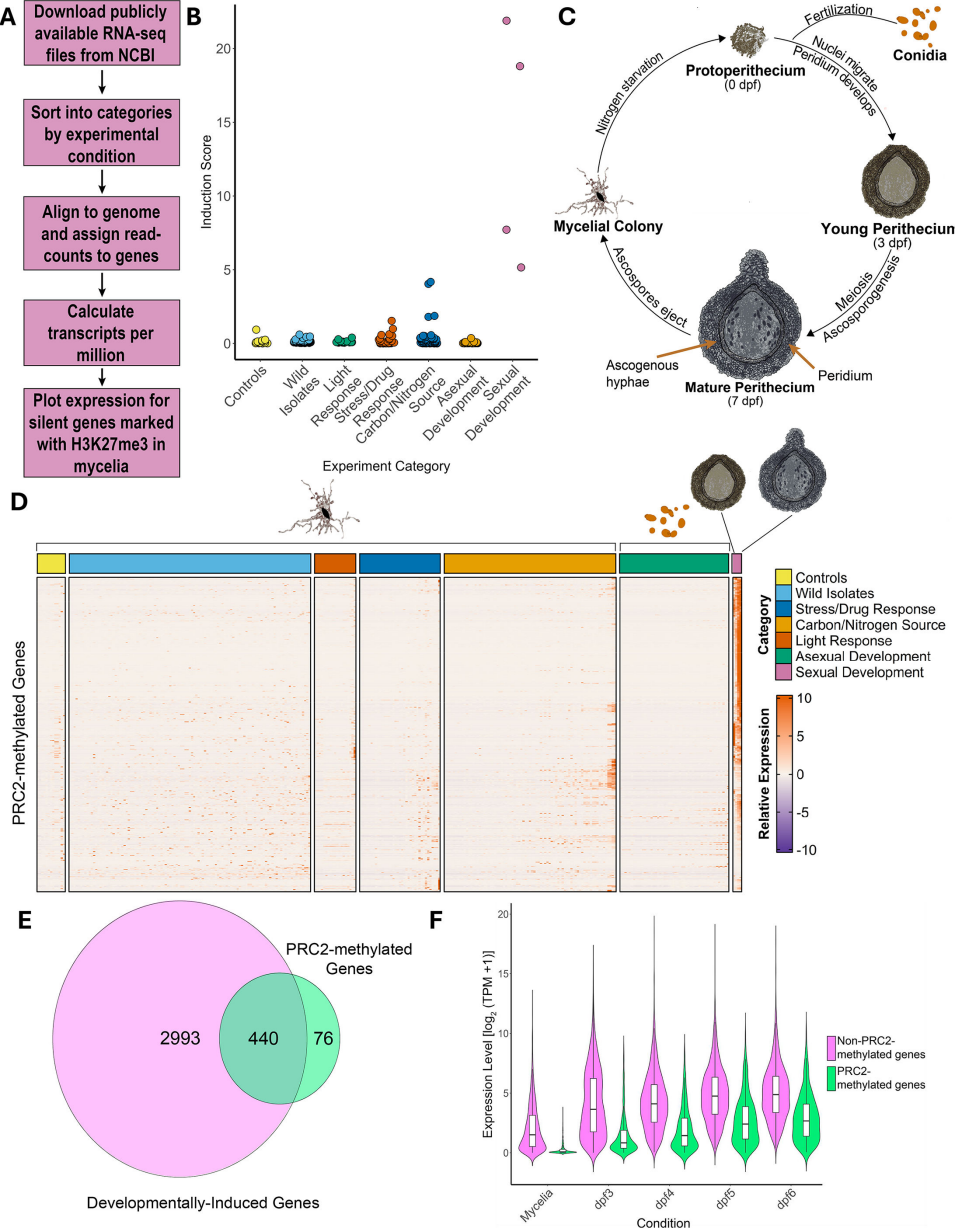
PRC2-methylated genes are broadly induced during sexual development. (**A**) The bioinformatics workflow used to examine expression of PRC2-methylated genes is shown. (**B**) The jitter plot shows the induction score of PRC2-methylated genes (*y*-axis) in each RNA-seq experiment (points). “Induction score” is the product of the mean expression of all PRC2-methylated genes in each experiment (TPM + 1) and the percentage of PRC2-methylated genes with a *z*-score greater than 0.5. (**C**) The schematic illustrates *N. crassa* sexual development. (**D**) The heatmap depicts relative expression of individual PRC2-methylated genes (rows; *n* = 516) in publicly available RNA-seq experiments (columns). (**E**) The Venn diagram shows overlap between genes induced during sexual development (l_2_fc > 2, FDR < 0.05; *n* = 2,993) and genes enriched for H3K27me3 (*n* = 516). (**F**) The violin plot depicts the expression level (log_2_[TPM + 1]) of DIGs that are enriched for H3K27me3 (green; *n* = 474) and DIGs that are not associated with H3K27me3 (magenta; *n* = 2,791) in mycelia or in developing perithecia isolated at 3, 4, 5, or 6-days post-fertilization (dpf).

Although PRC2-methylated genes are not expressed in mycelia grown under standard lab conditions (Vogel’s minimal medium + 1.5% sucrose) (7–9, 14), we hypothesized that PRC2-methylated genes are induced under specific conditions. Furthermore, we reasoned that identifying conditions in which PRC2-methylated genes are expressed could provide insight into the biological functions of PRC2 and PRC2-repressed genes. To test this, we obtained 428 publicly available RNA-seq experiments performed with wild-type “Oak Ridge” strains of *N. crassa* grown in a variety of nutritional conditions and at different stages of asexual or sexual development (strains 74-OR23-1V A or 4-OR8-1 a; Fig. 1A; Table S1). We then compared the relative expression of known PRC2-methylated genes in each condition (Table S2). Briefly, this list of PRC2-repressed genes was compiled by finding genes under H3K27me3 peaks in two independent ChIP-seq experiments and then filtering to remove expressed genes at peak boundaries (Supplemental Materials and Methods). For each experimental condition, we calculated a “gene induction score” for PRC2-methylated genes by multiplying the average expression level of this gene set by the percent of PRC2-methylated genes expressed (Fig. 1B). This analysis revealed a striking increase in transcript levels of PRC2-methylated genes under two experimental conditions: when Avicel was used as the sole carbon source, and during multiple stages of sexual (perithecial) development (Fig. 1B and C). We next plotted expression of individual PRC2-methylated genes in each experimental condition (Fig. 1D). Coordinated induction of PRC2-methylated genes occurred exclusively during perithecial development, though subsets of PRC2 target genes were weakly induced in other conditions such as treatment with cell wall-inhibiting drugs and growth on Avicel. Induction of PRC2-methylated genes during perithecial development was confirmed in a second RNA-seq data set (Fig. S1).

To further explore the relationship between perithecial development and PRC2-methylated genes, we performed differential expression analysis to identify all genes induced in developing perithecia (Tables S1; Supplemental Materials and Methods). We identified a total of 2,993 developmentally induced genes (DIGs), representing ~1/3 of the *N. crassa* genome. This set includes 85% (440/516) of H3K27me3-enriched genes (Fig. 1E; Table S3). Among PRC2-methylated genes, the DIGs were especially enriched for *Neurospora*-specific genes (Fig. S2a and b). Since most DIGs are not PRC2-methylated genes, we compared expression of PRC2-methylated DIGs and unmethylated DIGs across developmental stages (Fig. 1F). PRC2-methylated DIGs are strongly repressed in mycelial tissue, while DIGs that lack H3K27me3 are expressed in both mycelia and perithecia. We conclude that H3K27me3 marks a subset of perithecial-specific genes in *N. crassa*.

**Fig 2:**
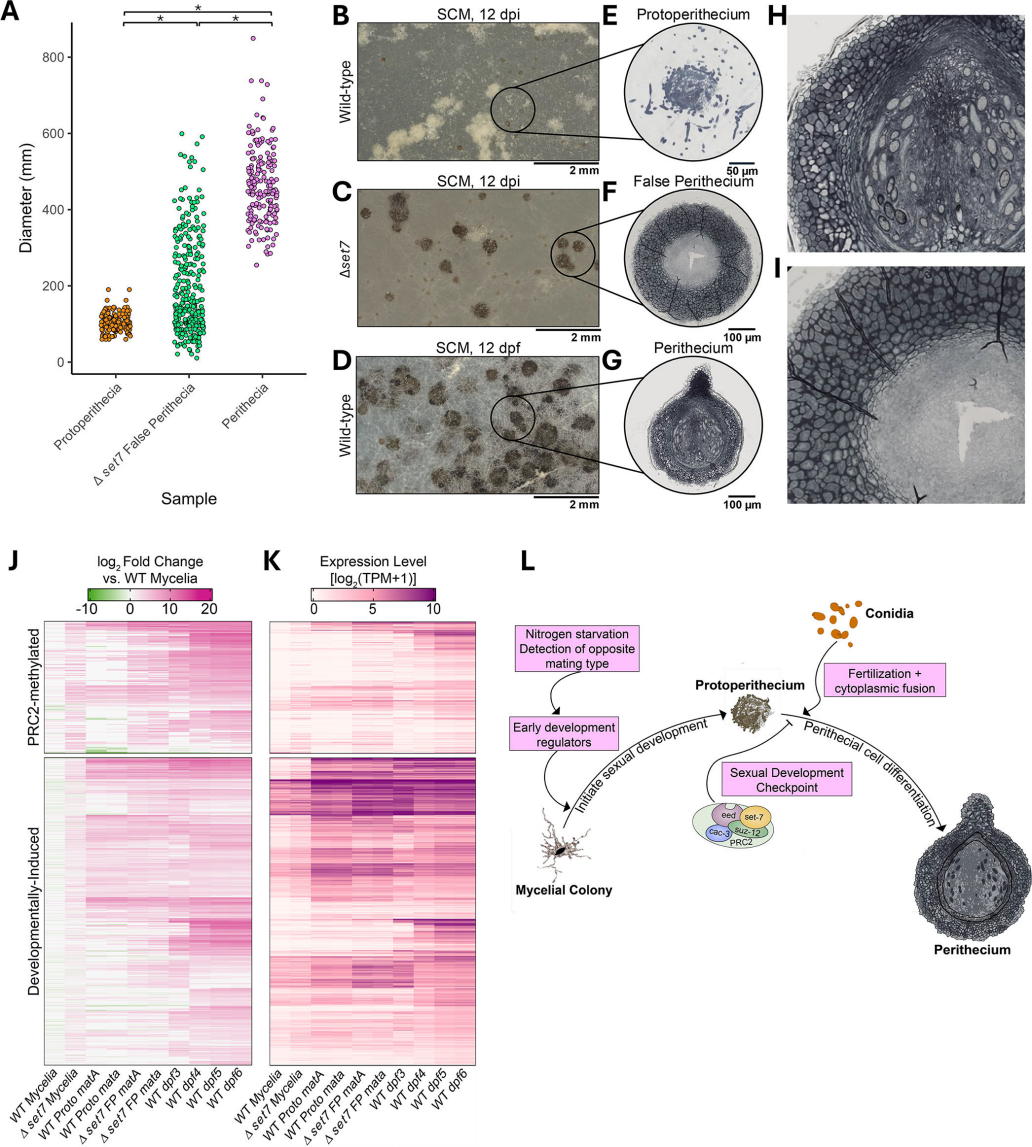
PRC2 is a repressor of perithecial development. (**A**) The points indicate the diameter (*y*-axis) of wild-type perithecia, wild-type protoperithecia, or ∆*set-7* false perithecia, as indicated (* = *P* < 0.001; Kruskal-Wallis test with Holm-adjusted Dunn *post hoc* test). (**B–D**) Representative images are shown for wild-type protoperithecia (unfertilized; 12 days post-inoculation), *Δset-7* false perithecia (unfertilized; 12 days post-inoculation), and wild-type perithecia (12 days post-fertilization) grown on SCM. Scale bars indicate 2 mm. (**E–G**) Representative images of paraffin-embedded, semi-thin (2.5 µm) sections of each structure from part A are shown. Sections were stained with toluidine blue before imaging using Bright Field microscopy. The scale bars indicate size in µm. (**H and I**) The magnified images of perithecia and false perithecia show distinct cell morphologies in the peridium. (**J**) The heatmaps illustrate relative expression levels (log_2_ fold change relative to mycelial samples) of PRC2-methylated (top) and non-PRC2-methylated DIGs (bottom) for the indicated samples. (**K**) Raw expression values (log_2_ [TPM + 1] are plotted as in panel J. (**L**) A model for PRC2-mediated regulation of sexual development is shown. PRC2 represses genes involved in the differentiation of perithecial cells until proper signals (fertilization) are received.

Because PRC2 directs the formation of repressed chromatin via deposition of H3K27me3, we hypothesized that PRC2 represses fruiting body development. Consistent with this idea, a previous study showed that PRC2-deficient mutants produce false perithecia, which were defined as large melanized structures that form in the absence of fertilization (11). To determine if the false perithecia produced in PRC2-deficient strains represent aberrant perithecial-like structures, we first characterized the numbers and sizes of melanized structures produced by the unfertilized ∆*set-7* mutant, which lacks the catalytic subunit of PRC2. We measured the diameter of melanized structures that formed on synthetic cross medium (SCM) plates with and without fertilization (Fig. 2A). In the absence of fertilization, the wild type produced protoperithecia with a relatively uniform diameter (~100 µm). After fertilization, the wild type produced highly melanized perithecia that were 400–600 µm in diameter. In contrast, the unfertilized ∆*set-7* strain produced large and highly melanized protoperithecia structures with sizes ranging from ~100 µm to 600µm, overlapping the sizes of both wild-type protoperithecia and fertilized perithecia.

To characterize the tissues within the aberrant structures produced by the unfertilized ∆*set-7* strain, we made semi-thin sections through wild-type protoperithecia, wild-type perithecia, and false perithecia from a *Δset-*7 strain, and we examined the morphology under the microscope (Fig. 2B through I). Wild-type perithecia are composed of maternally derived peridium tissues, which include the highly melanized outer wall, several inner wall layers, beak, and ostiole, as well as dikaryotic cell layers including the ascogonium and ascogenous hyphae (25–27). False perithecia produced by ∆*set-7* differentiated three cell layers: an outer layer of highly melanized tissue, a layer of elongated inner wall cells, and a layer of small, indistinct cells within the center. The two former cell layers resemble those found in the peridium of a wild-type perithecium (28). In contrast to wild-type perithecia, false perithecia produced by the ∆*set-7* strain lacked ascogenous hyphae, beaks, and ostioles. We conclude that loss of H3K27me3-mediated repression leads to aberrant development of the peridium, a phenotype resembling homeotic transformations observed in PRC2-deficient plant, insect, and animal cells. A similar developmental phenotype was previously observed in PRC2-deficient mutants of the fungus *Podospora anserina,* where loss of H3K27me3 triggers overproduction of male cells as well as abnormal perithecia (23). Together, these results show that PRC2 is a repressor of perithecial development.

To better understand transcriptional changes underlying this morphological transformation, we extracted RNA from ∆*set-7* strains of both mating types (mat A and mat a) grown overnight in liquid medium or on solid SCM for 12 days (Fig. S3a). On SCM plates, wild-type cells produce abundant protoperithecia (PP), and ∆*set-7* strains produce abundant false perithecia (FP). When ∆*set-7* was grown in liquid culture, a subset of H3K27me3-enriched genes exhibited weak upregulation (218/516, 42%) (Fig. 2J through K; Fig. S3b), consistent with prior studies (7). In contrast, a significant fraction of PRC2-methylated genes (336/516, 65%) were strongly upregulated in FP produced by *∆set-7* (Fig. 2J through K; Fig. S3b). These genes are typically expressed during perithecial development after fertilization has taken place (Fig. 2J through K). In the FP samples, the upregulation of DIGs was not restricted to PRC2-methylated genes, suggesting that global transcriptional reprogramming has occurred in these tissues (Fig. 2J through K; Fig. S3b).

In total, 1,391 genes demonstrated differential expression in false perithecia produced by ∆*set-7* strains but not in wild-type protoperithecia. Among these genes, we identified 44 transcription factors that belong to 10 different families (44/312, 14%; Fig. S4a and b) (29). Only four of these TFs also show changes in expression in *∆set-7* mycelia, and only one TF (NCU10501) is marked with H3K27me3 in mycelia. For most of these TFs, upregulation is much stronger in false perithecia than in any other condition (Fig. S4a and b). This includes several transcription factors required for normal development, such as *bek-2* and *vsd-9,* as well as the carbon catabolite repressor *cre-*1 (29, 30). We performed GO Term analysis to identify any other functional categories enriched in this data set (Table S4; Fig. S4c). Although many of the genes in this data set did not have an associated GO term, we found a statistically significant enrichment of terms relating to protein turnover and proteosome activity (Fig. S4c). Autophagy is critical for normal perithecial development, so it is possible that this process is also occurring in false perithecia (31).

We propose that PRC2 and its product, H3K27me3, form a developmental checkpoint in *N. crassa* (Fig. 2L). Although PRC2-methylated genes only represent a fraction of DIGs, these genes are notable for their exquisite cell-type specificity. PRC2-methylated DIGs are tightly repressed outside of development, while transcripts for most other DIGs are present in both mycelia and perithecia. Fungi must integrate multiple signals including nutritional stress and fusion with a partner of the opposite mating type to determine if differentiation of fruiting bodies is appropriate. Loss of PRC2 activity leads to aberrant development of false perithecia, which contain multiple tissue layers that comprise the maternally derived peridium. However, these structures are unable to develop late-arising maternal cell types that make up the beak and ostiole, or dikaryotic cell types that include the ascogonium and ascogenous hyphae. Fertilization to form a dikaryon and expression of both mating type genes is likely critical for the development of these structures. The false perithecia phenotype of PRC-deficient strains is remarkably similar to phenotypes of “self-stimulating” strains, where a pheromone precursor of the opposite mating type is expressed in a cell of the opposite mating type (32). Due to this similar phenotype, it is possible that PRC2 functions to block peridium development in the absence of pheromone-receptor binding to initiate fertilization. We conclude that PRC2 functions as a critical checkpoint component that ensures appropriate conditions for fruiting body development have been met before terminal differentiation is initiated.

## Data Availability

All RNA-sequencing data generated in this study are freely available at the NCBI Gene Expression Omnibus (https://www.ncbi.nlm.nih.gov/geo/) under the accession GSE296621. The code used for this analysis can be found on GitHub (https://github.com/UGALewisLab/2025_PRC2_Controls_Development).

## SUPPLEMENTAL MATERIAL

Supplemental Material mbio.01505-25-s0001.pdf Supplemental figures and methods.

Table S1 mbio.01505-25-s0002.xlsx RNA-seq experiments analyzed in this study.

Table S2 mbio.01505-25-s0003.txt PRC2-methylated genes.

Table S3 mbio.01505-25-s0004.xlsx Differential expression results.

Table S4 mbio.01505-25-s0005.txt GO terms.

ASM does not own the copyrights to Supplemental Material that may be linked to, or accessed through, an article. The authors have granted ASM a non-exclusive, world-wide license to publish the Supplemental Material files. Please contact the corresponding author directly for reuse.
