# Supplementary material for: Polycomb repressive complex 2 regulates sexual development in Neurospora crassa
Source: mBio. 2025 Sep 30;16(11):e01505-25. doi: 10.1128/mbio.01505-25 (PMC12607757; doi:10.1128/mbio.01505-25)
Supplement: Supplemental Material — Supplemental figures and methods. [file mbio.01505-25-s0001.pdf]

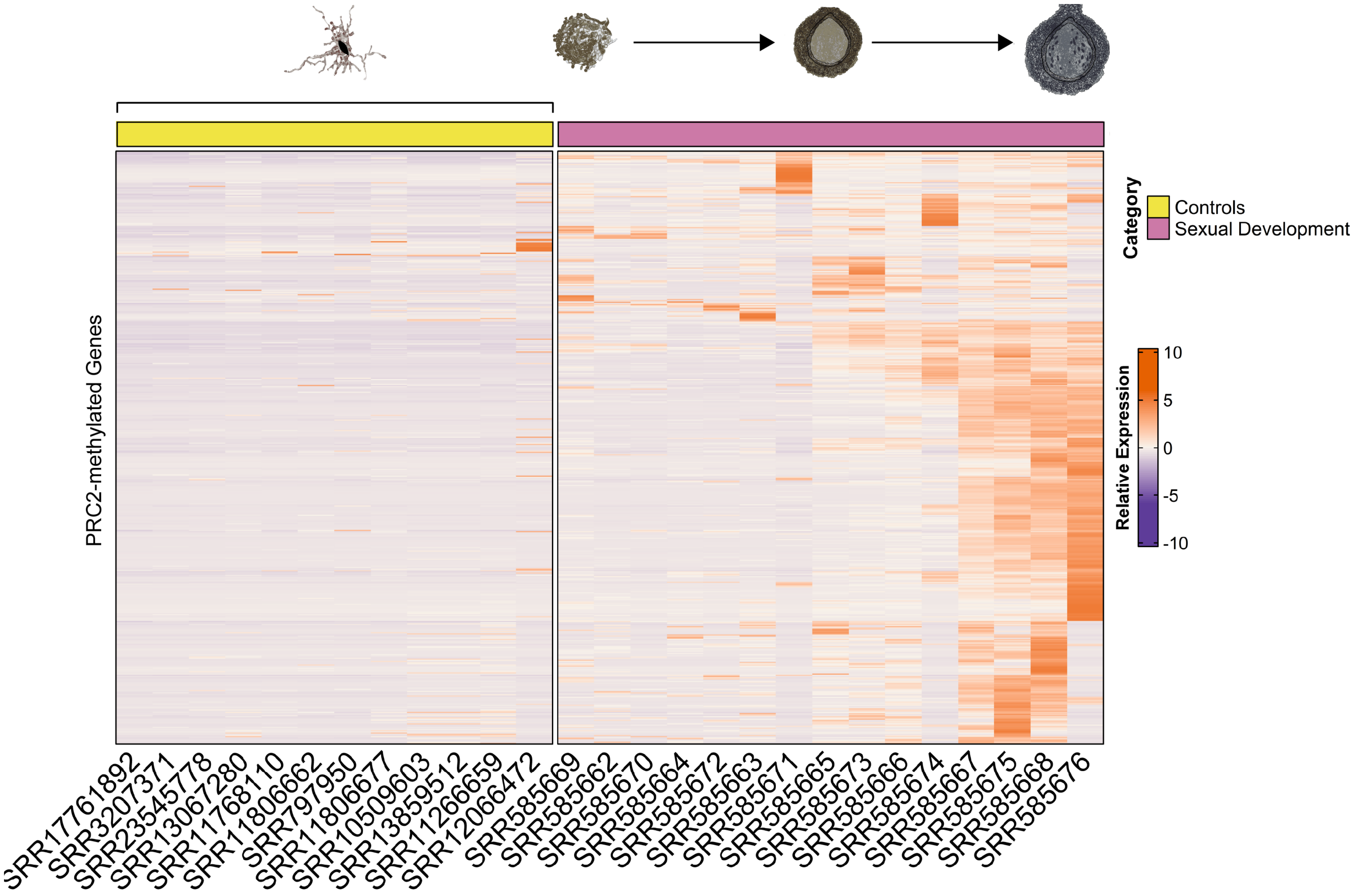

Figure S1. PRC2-methylated genes are upregulated in a second RNA-seq study of sexual development. The heatmap depicts relative expression of individual PRC2-methylated genes (rows; n = 516) in publicly available RNA-seq experiments (columns). Wild-type mycelial samples grown in standard laboratory conditions are shown under “Controls”, and RNA extracted from developing perithecia in another independent study are shown under “Sexual Development” (1). Samples are arranged by developmental stage (early development to late development from left to right; see Table S1 for details of each SRR).

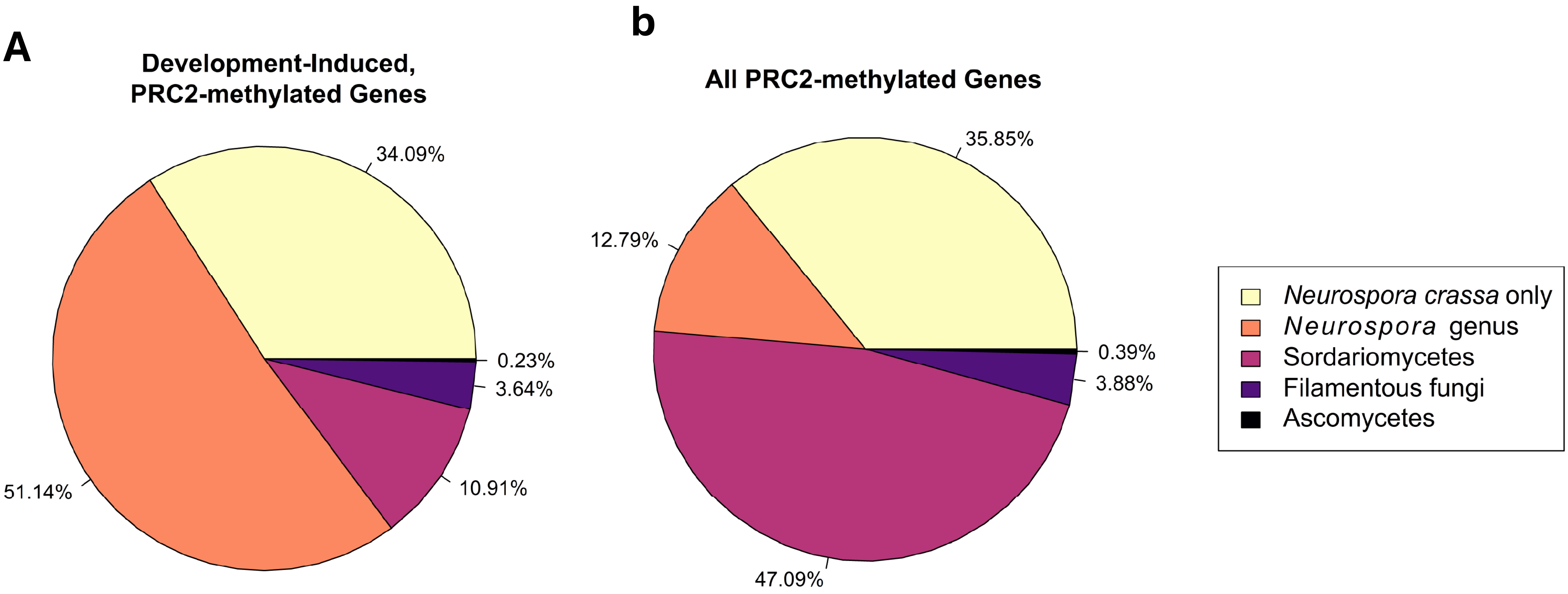

Figure S2. PRC2-methylated DIGs are enriched for *Neurospora*-specific genes. (A) The pie chart shows the percentage of PRC2-methylated DIGs (n=440) that have orthologs in each taxonomic group. (B) The pie chart shows that conservation of all PRC2-methylated genes (n=516) as in D.

A

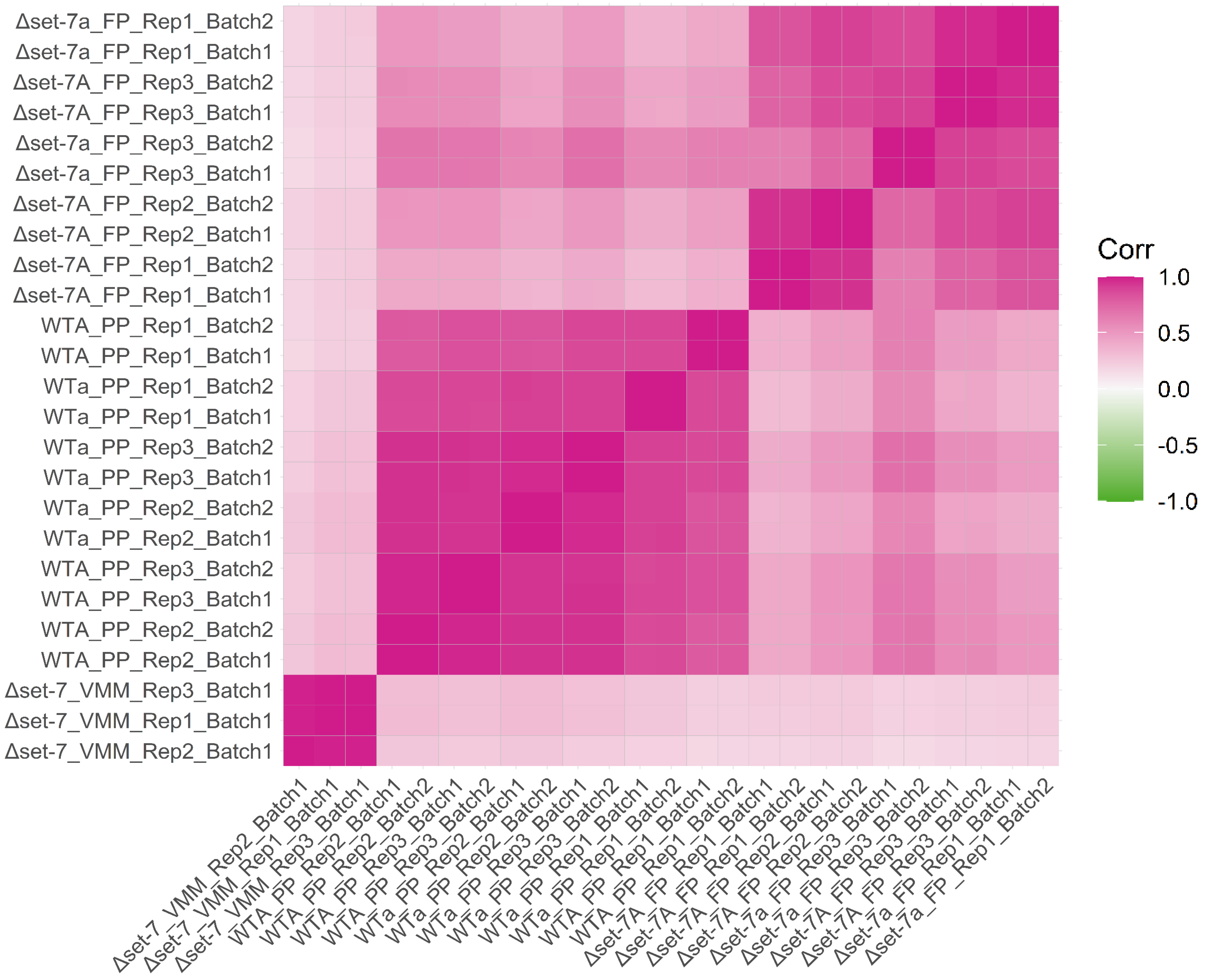

B

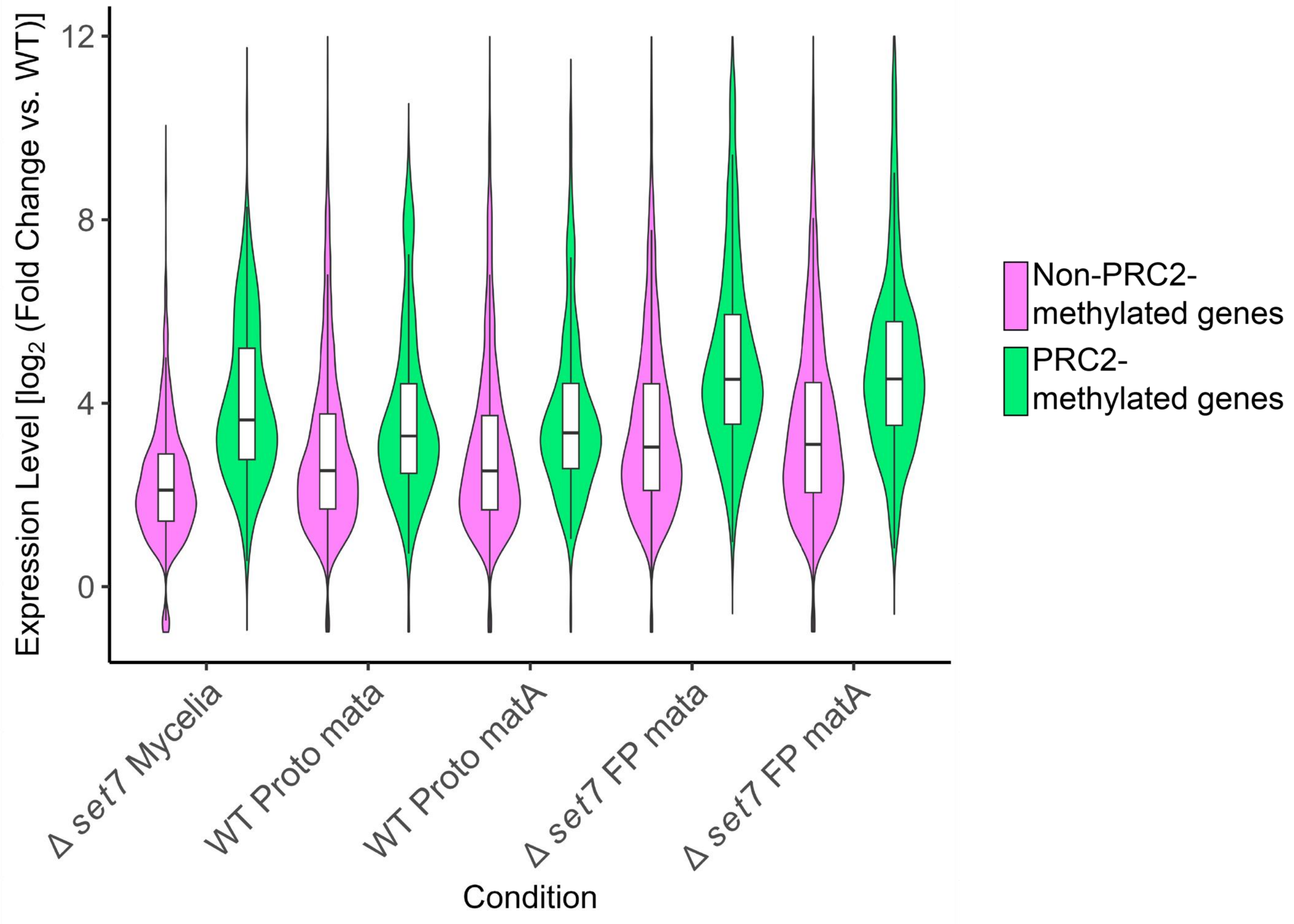

Figure S3. False perithecia demonstrate significant upregulation of both PRC2-methylated genes and general DIGs. (A) The correlation plot indicates that replicate RNA-seq samples are highly similar to one another. False perithecia cDNAs were sequenced in two batches to maximize read coverage. (B) The violin plot depicts the change in expression (log_2[Fold Change against WT mycelia] of DIGs that are enriched for H3K27me3 (green; n = 474) and DIGs that are not associated with H3K27me3 (magenta; n = 2,553) in RNA-seq profiles of *Δset-7* mycelia, WT protoperithecia, and *Δset-7* false perithecia.

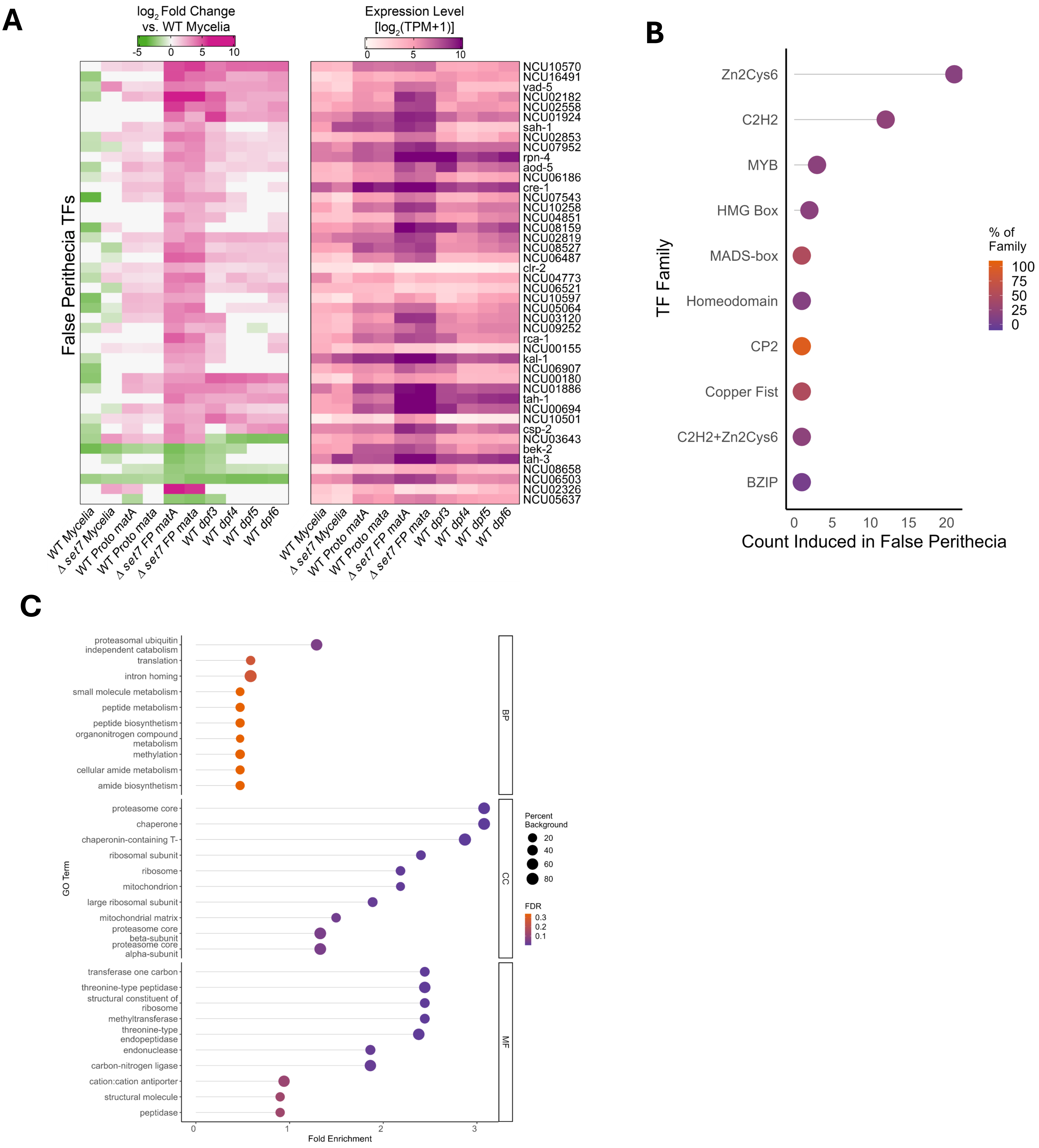

Figure S4. PRC2-methylated and false perithecia DIGs demonstrate unique properties. (A) The heatmaps demonstrate relative expression levels (left; log_2 fold change relative to mycelial samples) and raw expression levels (right; log_2[TPM+1]) of 44 transcriptions factors expressed at higher level sin in false perithecia compared to protoperithecia (B). The lollipop plot shows the number TFs in each family that are shown in A. The position of the circle represents how many members of the TF family are found in the dataset, and the color of the circle represents the percent of the TF family that is present. (C) The lollipop plot shows the fold enrichment of the top 10 most significant GO terms that are present in genes differentially expressed in false perithecia but not protoperithecia (n=1,391). Terms are divided by ontology (BP = Biological Process; CC = Cellular Component; MF = Molecular Function). The position of the circle represents the fold enrichment of the GO term within the FP dataset against the total number of genes related to that GO term (background) within the *N. crassa* genome. The size of the circle is proportional to the percentage of genes with that GO term in the dataset compared to the background, and the color of the circle is based on the Benjamini-Hochberg false discovery rate (FDR).

Materials & Methods

Strains and vegetative growth conditions

Wild-type strains of *Neurospora crassa* OR74A (FGSC2489; *mat A*) and 74-ORS-6a (FGSC4200; *mat a*) were obtained from the FGSC, and strains lacking individual PRC2 components were obtained from the *Neurospora crassa* knockout collection (www.fgsc.net) (2). Vegetative cultures were maintained on Vogel's Minimal Media + 1.5% sucrose agar slants (3). Crosses were conducted on Westergaard and Mitchell Synthetic Crossing Medium (SCM) supplemented with 1% sucrose and 2% agar, in petri dishes (4). All cross plates, except where otherwise noted, were maintained in constant darkness and ascospores were harvested no less than 14 days post-fertilization (dpf).

To obtain isogenic siblings for RNA-seq analysis, a *Δset-7* strain (FGSC11182, S238) was backcrossed to FGSC2489 (wild-type; S2). The following progeny were isolated and used for analysis: S890 (*Δset-7* mat A), S892 (*Δset-7* mat a), S894 (wild-type mat A), S896 (wild-type mat A).

False Perithecia Microscopy and Sectioning

False perithecia were obtained by inoculating 20,000 cells into the center of an SCM plate, which was then incubated in constant darkness for 7-14 days. Images were obtained at 7 and 14 days post-inoculation (dpi) using a Keyence VHX-600 microscope. Diameters of perithecia were measured using ImageJ.

To obtain images of mature perithecia, developing protoperithecia, and false perithecia, structures were scraped from an agar plate at 12 dpi (for protoperithecia and

false perithecia) or 10 dpf (for perithecia). Wild-type protoperithecia were obtained by cutting 1 cm disks using a cork borer and gently dissecting away excess agar. Harvested samples were washed once with PBS and then fixed for 48 hours in a solution of 2% glutaraldehyde + 1.9% formaldehyde in 0.025M phosphate buffer, as previously described (5). Samples were embedded in paraffin and cut into 2.5 µm semithin sections and stained with 1% toluidine blue. Sections were examined using an Echo Revolve microscope, with Olympus UPlanXApo 20x / 0.80 and 60x / 1.42 Oil lenses.

Identification of PRC2-methylated genes

To generate a consensus list of genes regulated by PRC2, publicly available H3K27me2/3 and H3K36me3 ChIP-seq datasets performed in wild-type *N. crassa* mycelia were downloaded from the SRA (Input: SRR7690277; H3K27me2/3: SRR7690281, SRR12229314; H3K36me3: SRR12229309, SRR7690285) (6, 7). Illumina adaptors were trimmed using Trim_Galore (0.6.7), aligned to the *N. crassa* genome using BWA (0.7.17), and indexed using SAMtools (1.16.1). H3K27me3 and H3K36me3 peaks were called using MACS3 (3.0.0b1) with parameters optimized for broad histone peaks. Peak calls were intersected with *N. crassa* genes using the Intersect command from BEDTools, with a gene coverage threshold of 50%. To identify a consensus peakset, only peaks that occurred in all four ChIP-seq datasets were used. Expressed genes at the boundary of PRC2 methylated domains were removed from the consensus peakset by calculating the median TPM of PRC2-methylated genes in all “control” datasets, and then identifying statistical outliers using the interquartile range

(IQR) approach. The final list of PRC2-methylated genes used for analysis can be found in Table S3.

The conservation of PRC2-methylated DIGs and non-DIGs was assessed using OrthoMCL-DB (8). As described previously, orthologs of each PRC2-methylated gene were identified in representative organisms within the *Neurospora* genus (*N. discreta and N. tetrasperma*), Sordariomycetes (*Sordaria macrospora* and *Podospora anserina*), filamentous fungi (*Aspergillus fumigatus, Zymoseptoria tritici,* and *Pyronema omphalodes*), and the Ascomycetes (*Saccharomyces cerevisiae* and *Schizosaccharomyces pombe*) (9).

RNA Isolation, Sequencing, and Analysis

RNA was extracted from false perithecia following protocols adapted from previous RNA-seq experiments conducted in perithecia (1, 10). Briefly, isogenic siblings were isolated from a backcross between FGSC11182 (*Δset-7*) and wild-type *N. crassa.* Individual wild-type and *Δset-7* progeny were inoculated on plates containing SCM agar overlain with cellophane and grown at room temperature in constant darkness for 12 days. Tissue was harvested from the cellophane surface and then snap frozen and ground in liquid nitrogen. RNA was isolated using TRIzol reagent, using methods optimized for fungal tissue isolated from solid surfaces (11). cDNA libraries were prepared using the NEBNext Poly(A) mRNA Magnetic Isolation kit (NEB #E7490) and NEBNext Ultra II Directional RNA Library Prep Kit for Illumina.

Publicly available RNA-seq data was obtained from the NCBI SRA. For all RNA-seq experiments, Illumina adaptors were trimmed using Trim_Galore (0.6.7) and aligned

to the *N. crassa* genome with STAR (2.7.10b). To visualize expression dynamics, readcounts per gene were compiled using Subread (2.0.6), which were subsequently used to calculate transcripts per million using the Scater package (1.30.1) and then pseduocounts (TPM + 1). To identify differentially expressed genes during sexual development, raw counts were passed to edgeR (4.0.16), and genes were filtered to remove artefacts from lowly-expressed genes (less than 5 counts per gene in total). The full list of SRA datasets used for this analysis can be found in Table S1.

Differential expression was calculated against an aggregate of wild-type mycelial RNA-seq samples that clustered closely together based on a correlation plot generated by the corrplot package (0.95). To control for variation between RNA-seq datasets, we included two separate wild type RNA-seq files in the differential expression analysis (SRR177529 and SRR17730).

We identified genes that demonstrated statistically significant differential expression ($\log_2$(Fold Change vs. Mycelia) < -2 or > 2, FDR < 0.05) in false perithecia but not in wild-type protoperithecia. These genes were compared to a previously published list of *N. crassa* transcription factors to identify differentially-expressed TFs and their TF families (12). We also performed differential Gene Ontology enrichment on these genes using FungiDB (13, 14).

While analyzing the RNA-seq data generated from false perithecia, we determined that all sequencing samples included high coverage of a contaminating PCR product corresponding to the promoter region and 5’ coding sequence of NCU08634. To avoid bias in the data, this region was masked prior to readcount compilation.

Supplementary References

1. Wang Z, Lopez-Giraldez F, Lehr N, Farré M, Common R, Trail F, Townsend JP. 2014. Global gene expression and focused knockout analysis reveals genes associated with fungal fruiting body development in Neurospora crassa. Eukaryot Cell 13:154–169.

2. Colot H V., Park G, Turner GE, Ringelberg C, Crew CM, Litvinkova L, Weiss RL, Borkovich KA, Dunlap JC. 2006. A high-throughput gene knockout procedure for Neurospora reveals functions for multiple transcription factors. Proc Natl Acad Sci U S A 103:10352–10357.

3. Davis RH, de Serres FJ. 1970. [4] Genetic and microbiological research techniques for Neurospora crassa. Methods Enzymol 17:79–143.

4. Westergaard M, Mitchell HK. 1947. Neurospora V. A Synthetic Medium Favoring Sexual Reproduction. Am J Bot 34:573–577.

5. Trail F, Common R. 2000. Perithecial development by Gibberella zeae: A light microscopy study. Mycologia 92:130–138.

6. Ferraro AR, Ameri AJ, Lu Z, Kamei M, Schmitz RJ, Lewis ZA. 2021. Chromatin accessibility profiling in Neurospora crassa reveals molecular features associated with accessible and inaccessible chromatin. BMC Genomics 22.

7. Bicocca VT, Ormsby T, Adhvaryu KK, Honda S, Selker EU. 2018. ASH1-catalyzed H3K36 methylation drives gene repression and marks H3K27me2/3-competent chromatin. Elife 7.

8. Chen F, Mackey AJ, Stoeckert CJ, Roos DS. 2006. OrthoMCL-DB: querying a comprehensive multi-species collection of ortholog groups. Nucleic Acids Res 34.

9. Jamieson K, Rountree MR, Lewis ZA, Stajich JE, Selker EU. 2013. Regional control of histone H3 lysine 27 methylation in Neurospora. Proc Natl Acad Sci U S A 110:6027–6032.

10. Liu H, Li Y, Chen D, Qi Z, Wang Q, Wang J, Jiang C, Xu JR. 2017. A-to-I RNA editing is developmentally regulated and generally adaptive for sexual reproduction in Neurospora crassa. Proc Natl Acad Sci U S A 114:E7756–E7765.

11. Schumann U, Smith NA, Wang MB. 2013. A fast and efficient method for preparation of high-quality RNA from fungal mycelia. BMC Res Notes 6:1–5.

12. Carrillo AJ, Schacht P, Cabrera IE, Blahut J, Prudhomme L, Dietrich S, Bekman T, Mei J, Carrera C, Chen V, Clark I, Fierro G, Ganzen L, Orellana J, Wise S, Yang K, Zhong H, Borkovich KA. 2017. Functional Profiling of Transcription Factor Genes in Neurospora crassa. G3 (Bethesda) 7:2945–2956.

13. Consortium TGO, Aleksander SA, Balhoff J, Carbon S, Cherry JM, Drabkin HJ, Ebert D, Feuermann M, Gaudet P, Harris NL, Hill DP, Lee R, Mi H, Moxon S, Mungall CJ, Muruganugan A, Mushayahama T, Sternberg PW, Thomas PD, Van Auken K, Ramsey J, Siegele DA, Chisholm RL, Fey P, Aspromonte MC, Nugnes MV, Quaglia F, Tosatto S, Giglio M, Nadendla S, Antonazzo G, Attrill H, dos Santos G, Marygold S, Strelets V, Tabone CJ, Thurmond J, Zhou P, Ahmed SH, Asanitthong P, Luna Buitrago D, Erdol MN, Gage MC, Ali Kadhum M, Li KYC, Long M, Michalak A, Pesala A, Pritazahra A, Saverimuttu SCC, Su R, Thurlow KE, Lovering RC, Logie C, Oliferenko S, Blake J, Christie K, Corbani L, Dolan ME, Drabkin HJ, Hill DP, Ni L, Sitnikov D, Smith C, Cuzick A, Seager J, Cooper L, Elser J, Jaiswal P, Gupta P, Jaiswal P, Naithani S, Lera-Ramirez M, Rutherford K, Wood V, De Pons JL, Dwinell MR, Hayman GT, Kaldunski ML, Kwitek AE, Laulederkind SJF, Tutaj MA, Vedi M, Wang S-J, D'Eustachio P, Aimo L, Axelsen K, Bridge A, Hyka-Nouspikel N, Morgat A, Aleksander SA, Cherry JM, Engel SR, Karra K, Miyasato SR, Nash RS, Skrzypek MS, Weng S, Wong ED, Bakker E, Berardini TZ, Reiser L, Auchincloss A, Axelsen K, Argoud-Puy G, Blatter M-C, Boutet E, Breuza L, Bridge A, Casals-Casas C, Coudert E, Estreicher A, Livia Famiglietti M, Feuermann M, Gos A, Gruaz-Gumowski N, Hulo C, Hyka-Nouspikel N, Jungo F, Le Mercier P, Lieberherr D, Masson P, Morgat A, Pedruzzi I, Pourcel L, Poux S, Rivoire C, Sundaram S, Bateman A, Bowler-Barnett E, Bye-A-Jee H, Denny P, Ignatchenko A, Ishtiaq R, Lock A, Lussi Y, Magrane M, Martin MJ, Orchard S, Raposo P, Speretta E, Tyagi N, Warner K, Zaru R, Diehl AD, Lee R, Chan J, Diamantakis S, Raciti D, Zarowiecki M, Fisher M, James-Zorn C, Ponferrada V, Zorn A, Ramachandran S, Ruzicka L, Westerfield M, Aleksander SA, Balhoff J, Carbon S, Cherry JM, Drabkin HJ, Ebert D, Feuermann M, Gaudet P, Harris NL, Hill DP, Lee R, Mi H, Moxon S, Mungall CJ, Muruganugan A, Mushayahama T, Sternberg PW, Thomas PD, Van Auken K, Ramsey J, Siegele DA, Chisholm RL, Fey P, Aspromonte MC, Nugnes MV, Quaglia F, Tosatto S, Giglio M, Nadendla S, Antonazzo G, Attrill H, dos Santos G, Marygold S, Strelets V, Tabone CJ, Thurmond J, Zhou P, Ahmed SH, Asanitthong P, Luna Buitrago D, Erdol MN, Gage MC, Ali Kadhum M, Li KYC, Long M, Michalak A, Pesala A, Pritazahra A, Saverimuttu SCC, Su R, Thurlow KE, Lovering RC, Logie C, Oliferenko S, Blake J, Christie K, Corbani L, Dolan ME, Drabkin HJ, Hill DP, Ni L, Sitnikov D, Smith C, Cuzick A, Seager J, Cooper L, Elser J, Jaiswal P,

Gupta P, Jaiswal P, Naithani S, Lera-Ramirez M, Rutherford K, Wood V, De Pons JL, Dwinell MR, Hayman GT, Kaldunski ML, Kwitek AE, Laulederkind SJF, Tutaj MA, Vedi M, Wang S-J, D'Eustachio P, Aimo L, Axelsen K, Bridge A, Hyka-Nouspikel N, Morgat A, Aleksander SA, Cherry JM, Engel SR, Karra K, Miyasato SR, Nash RS, Skrzypek MS, Weng S, Wong ED, Bakker E, Berardini TZ, Reiser L, Auchincloss A, Axelsen K, Argoud-Puy G, Blatter M-C, Boutet E, Breuza L, Bridge A, Casals-Casas C, Coudert E, Estreicher A, Livia Famiglietti M, Feuermann M, Gos A, Gruaz-Gumowski N, Hulo C, Hyka-Nouspikel N, Jungo F, Le Mercier P, Lieberherr D, Masson P, Morgat A, Pedruzzi I, Pourcel L, Poux S, Rivoire C, Sundaram S, Bateman A, Bowler-Barnett E, Bye-A-Jee H, Denny P, Ignatchenko A, Ishtiaq R, Lock A, Lussi Y, Magrane M, Martin MJ, Orchard S, Raposo P, Speretta E, Tyagi N, Warner K, Zaru R, Diehl AD, Lee R, Chan J, Diamantakis S, Raciti D, Zarowiecki M, Fisher M, James-Zorn C, Ponferrada V, Zorn A, Ramachandran S, Ruzicka L, Westerfield M. 2023. The Gene Ontology knowledgebase in 2023. Genetics 224.

14. Alvarez-Jarreta J, Amos B, Aurrecoechea C, Bah S, Barba M, Barreto A, Basenko EY, Belnap R, Blevins A, Böhme U, Brestelli J, Brown S, Callan D, Campbell LI, Christophides GK, Crouch K, Davison HR, DeBarry JD, Demko R, Doherty R, Duan Y, Dundore W, Dyer S, Falke D, Fischer S, Gajria B, Galdi D, Giraldo-Calderón GI, Harb OS, Harper E, Helb D, Howington C, Hu S, Humphrey J, Iodice J, Jones A, Judkins J, Kelly SA, Kissinger JC, Kittur N, Kwon DK, Lamoureux K, Li W, Lodha D, MacCallum RM, Maslen G, McDowell MA, Myers J, Nural MV, Roos DS, Rund SSC, Shanmugasundram A, Sitnik V, Spruill D, Starns D, Tomko SS, Wang H, Warrenfeltz S, Wieck R, Wilkinson PA, Zheng J. 2024. VEuPathDB: the eukaryotic pathogen, vector and host bioinformatics resource center in 2023. Nucleic Acids Res 52:D808–D816.
